# Pré-Condicionamento na Lesão por Isquemia-Reperfusão

**DOI:** 10.36660/abc.20210908

**Published:** 2021-11-22

**Authors:** Mariana Gatto, Gustavo Augusto Ferreira Mota, Luana Urbano Pagan, Mariana Janini Gomes, Marina Politi Okoshi

**Affiliations:** 1 Universidade Estadual Paulista Faculdade de Medicina de Botucatu Departamento de Clínica Médica Botucatu SP Brasil Departamento de Clínica Médica - Faculdade de Medicina de Botucatu - Universidade Estadual Paulista (UNESP), Botucatu, SP – Brasil; 2 Harvard Medical School Brigham and Women's Hospital Boston MA EUA Brigham and Women's Hospital, Harvard Medical School, Boston, MA – EUA

**Keywords:** Condicionamento Físico Animal, Tratamento Farmacológico, Reperfusão Miocárdica, Isquemia Miocárdica, Apoptose, Ratos

A cardiopatia isquêmica é caracterizada pela diminuição do fluxo sanguíneo miocárdico, que reduz a relação entre oferta e demanda de oxigênio. A condição atrai a atenção da comunidade científica devido a sua grande incidência, elevada morbidade e mortalidade, e alto custo terapêutico.^1^ O tratamento envolve, primariamente, a revascularização miocárdica. Paradoxalmente, a reperfusão e restabelecimento do fluxo sanguíneo podem causar dano adicional, chamado de injúria miocárdica da isquemia-reperfusão (IR).^2^ Muitos mecanismos estão implicados na injúria da IR como, por exemplo, alterações na homeostase iônica, desajuste no trânsito intracelular de cálcio, disfunção metabólica e mitocondrial, e aumento da inflamação e da produção de espécies reativas do oxigênio.^3,4^ Esses fatores podem contribuir para o aumento da área de necrose miocárdica, do desenvolvimento de insuficiência cardíaca pós-isquemia, e do risco de morte.^3^

Em meados da década de 80, foi descrito o pré-condicionamento isquêmico, que consiste na aplicação de curtos períodos de isquemia coronária alternados com reperfusão; quando antecede período isquêmico maior, o pré-condicionamento isquêmico reduz o tamanho do infarto.^5,6^ O efeito benéfico do pré-condicionamento isquêmico foi demonstrado em pacientes acometidos por angina; quando os mesmos evoluíram para infarto do miocárdio, apresentaram menor tamanho da área infartada e melhor evolução clínica que pacientes assintomáticos antes do infarto.^7,8^ Adicionalmente, o pré-condicionamento de ratos com estresse físico agudo anteriormente ao período de isquemia e reperfusão reduziu o tamanho do infarto e melhorou os parâmetros hemodinâmicos.^9^

Atualmente, métodos de isquemia-reperfusão têm sido utilizados experimentalmente para avaliar se diferentes terapias farmacológicas e não farmacológicas são efetivas em proteger o coração de eventos isquêmicos e da reperfusão. Entretanto, investigações conduzidas com fármacos como atorvastatina, anti-inflamatórios, ou antioxidantes não resultaram em cardioproteção após isquemia miocárdica.^10^ O ensaio clínico multicêntrico CIRCUS avaliou os efeitos da administração de ciclosporina A antes de intervenção percutânea para revascularização de pacientes com infarto agudo do miocárdio. Embora tenha ocorrido redução da área infartada, não houve melhora clínica a longo prazo.^11^ Atualmente, não existem fármacos específicos para prevenir ou atenuar a lesão da IR.

A dexmedetomidina (DEX) é um agonista do receptor α2-adrenérgico utilizado na prática clínica para induzir, principalmente, analgesia e sedação. Em estudo experimental, o pré-condicionamento com DEX melhorou a função ventricular esquerda de ratos.^12^ Entretanto, os mecanismos moleculares relacionados com a cardioproteção induzida pela DEX ainda não estão completamente esclarecidos. Nesta edição dos Arquivos Brasileiros de Cardiologia, lemos com grande interesse o estudo de Chen et al.,^13^ que avaliaram os efeitos protetores da administração de DEX previamente à isquemia-reperfusão do miocárdio de ratos. O tratamento com DEX melhorou variáveis hemodinâmicas e de função cardíaca e atenuou a área infartada em comparação aos animais não tratados. A melhora cardíaca foi relacionada com redução de apoptose miocárdica, constatada por análises morfológicas e pela inibição da expressão de proteínas da via apoptótica PERK/eIF2α/TCF-4/CHOP. Os autores também observaram redução da expressão da proteína GRP78, importante marcadora de estresse do retículo endoplasmático.

Os resultados são instigantes e mostram a necessidade de estudos adicionais para melhor esclarecer os efeitos moleculares da dexmedetomidina na cardioproteção após lesão miocárdica induzida por isquemia seguida de reperfusão.
