# Supplementary material for: Pré-Condicionamento na Lesão por Isquemia-Reperfusão
Source: Arq Bras Cardiol. 2021 Nov 22;117(6):1145–6. [Article in Portuguese] doi: 10.36660/abc.20210908 (PMC8757153; doi:10.36660/abc.20210908)
Supplement: Translation

# Translation: Preconditioning in Ischemia-Reperfusion Lesion

**Authors:** Mariana Gatto, Gustavo Augusto Ferreira Mota, Luana Urbano Pagan, Mariana Janini Gomes, Marina Politi Okoshi

**Affiliations:** 1 Botucatu SP Brazil Departamento de Clínica Médica - Faculdade de Medicina de Botucatu - Universidade Estadual Paulista (UNESP), Botucatu, SP – Brazil; 2 Boston MA USA Brigham and Women's Hospital, Harvard Medical School, Boston, MA – USA

**Keywords:** Physical Conditioning, Drug Therapy, Animal, Myocardial Reperfusion, Myocardial Ischemia, Apoptosis, Rats

Ischemic heart disease is characterized by a decrease in myocardial blood flow, which reduces the relationship between oxygen supply and demand. The condition has attracted the attention of the scientific community due to its high incidence, elevated morbidity and mortality, and high therapeutic cost.^1^ The treatment primarily relies on myocardial revascularization. Paradoxically, reperfusion and restoration of blood flow can cause additional damage, called myocardial ischemia-reperfusion (IR) injury.^2^ Several mechanisms are involved in the IR injury, such as ionic homeostasis change, intracellular calcium transient alteration, metabolic and mitochondrial dysfunction, inflammation, and reactive oxygen species increase.^3,4^ These factors may contribute to an increase in the myocardial necrosis size, post-ischemia heart failure, and death.^3^

First identified in the mid-1980s, ischemic preconditioning is a process whereby repeated application of short periods of ischemia alternating with reperfusion protect the myocardium from longer ischemic insults, therefore reducing the infarction size.^5,6^ The beneficial effect of ischemic preconditioning was shown in angina patients; when evolving to myocardial infarction, they had a smaller infarcted area and a better clinical prognosis than previously asymptomatic patients.^7,8^ Additionally, preconditioning of rats with acute physical stress prior to the IR insult reduced infarct size and improved hemodynamic parameters.^9^

Currently, methods to induce ischemia-reperfusion have been used experimentally to assess whether different pharmacological and non-pharmacological therapies are effective in protecting the heart from ischemia and reperfusion injury. However, investigations to evaluate potential drugs, such as atorvastatin, anti-inflammatory medicines, or antioxidants did not result in cardioprotection after myocardial ischemia.^10^ The CIRCUS multicenter clinical trial evaluated the effects of cyclosporine A administration before revascularization with percutaneous coronary intervention in acute myocardial infarction patients. Despite a reduction in the infarcted area, there was no long-term clinical improvement.^11^ Currently, there are no specific drugs to prevent or attenuate IR injury.

Dexmedetomidine (DEX) is an α2-adrenergic receptor agonist used in clinical practice mainly to induce analgesia and sedation. Preconditioning with DEX improved left ventricular function in rats.^12^ However, molecular mechanisms related to DEX-induced cardioprotection are still not fully understood. In this edition of ABC, we read with great interest the study by Chen et al.^13^ evaluating the protective effects of DEX administration prior to myocardial ischemia-reperfusion in rats. DEX treatment improved hemodynamic and cardiac function variables and attenuated the infarcted area compared to the untreated animals. Cardiac improvement was combined with reduced myocardial apoptosis, assessed by morphological analysis, and inhibited expression of proteins of the apoptotic pathway PERK/eIF2α/TCF-4/CHOP. The authors also observed a decrease in the expression of the GRP78 protein, an important marker of endoplasmic reticulum stress.

The results are exciting and show the need for additional studies to better clarify the molecular effects of dexmedetomidine on cardioprotection after myocardial injury induced by ischemia followed by reperfusion.
